# Vicious circle between progressive right ventricular dilatation and pulmonary regurgitation in patients after tetralogy of Fallot repair? Right heart enlargement promotes flow reversal in the left pulmonary artery

**DOI:** 10.1186/s12968-016-0254-1

**Published:** 2016-06-07

**Authors:** Atsuko Kato, Christian Drolet, Shi-Joon Yoo, Andrew N. Redington, Lars Grosse-Wortmann

**Affiliations:** Division of Cardiology, Department of Paediatrics, The Hospital for Sick Children, University of Toronto, 555 University Ave, Toronto, M5G 1X8 ON Canada; now: Université Laval, Quebec City, QC Canada; Department of Diagnostic Imaging, The Hospital for Sick Children, University of Toronto, Toronto, ON Canada; now: Heart Institute, Cincinnati Children’s Hospital, Cincinnati, OH USA

**Keywords:** Differential pulmonary blood flow, Pulmonary regurgitation, Tetralogy of Fallot, Right ventricular enlargement, Cardiovascular magnetic resonance

## Abstract

**Background:**

The left pulmonary artery (LPA) contributes more than the right (RPA) to total pulmonary regurgitation (PR) in patients after tetralogy of Fallot (TOF) repair, but the mechanism of this difference is not well understood. This study aimed to analyze the interplay between heart and lung size, mediastinal geometry, and differential PR.

**Methods:**

Forty-eight Cardiovascular Magnetic Resonance (CMR) studies in patients after TOF repair were analyzed. In addition to the routine blood flow and ventricular volume quantification cardiac angle between the thoracic anterior-posterior line and the interventricular septum, right and left lung areas as well as right and left hemithorax areas were measured on an axial image. Statistical analysis was performed to compare flow parameters between RPA and LPA and to assess correlation among right ventricular volume, pulmonary blood flow parameters and lung area.

**Results:**

There was no difference between LPA and RPA diameters. The LPA showed significantly less total forward flow (2.49 ± 0.87 L/min/m^2^ vs 2.86 ± 0.89 L/min/m^2^; *p* = 0.02), smaller net forward flow (1.40 ± 0.51 vs 1.89 ± 0.60 mL/min/m^2^; *p* = <0.001), and greater regurgitant fraction (RF) (34 ± 10 % vs 43 ± 12 %; *p* = 0.001) than the RPA. There was no difference in regurgitant flow volume between RPA and LPA (*p* = 0.29). Indexed right ventricular end-diastolic volume (RVEDVi) correlated with LPA RF (*R* = 0.48, *p* < 0.001), but not with RPA RF (*p* = 0.09). Larger RVEDVi correlated with a more leftward cardiac axis (*R* = 0.46, *p* < 0.001) and with smaller left lung area (*R* = −0.58, *p* < 0.001). LPA RF, but not RPA RF, correlated inversely with left lung area (*R* = −0.34, *p* = 0.02). The follow-up CMRs in 20 patients showed a correlation of the rate of RV enlargement with the rates of LPA RF worsening (*R* = 0.50, *p* = 0.03), and of increasing left lung compression (*R* = −0.55, *p* = 0.012).

**Conclusion:**

An enlarged and levorotated heart is associated with left lung compression and impaired flow into the left lung.

## Background

Tetralogy of Fallot (TOF) is the most common cyanotic congenital heart disease. Early postoperative outcomes after closure of the ventricular septal defect and reconstruction of the right ventricular outflow tract are excellent in the majority of patients [1]. However, chronic pulmonary regurgitation (PR) after TOF repair is a major determinant of long-term outcomes. Right ventricular (RV) dilation, resulting from PR, is associated with exercise intolerance, ventricular arrhythmia, and mortality [2–4]. Despite a wealth of evidence for the association of PR and RV dilation, the driving forces behind PR remain incompletely understood [5–8].

Cardiovascular Magnetic Resonance (CMR) is the gold standard for the quantification of PR and RV volume [9–12]. Using phase-contrast CMR, we previously demonstrated that PR is greater in the left pulmonary artery (LPA) than in the right pulmonary artery (RPA) in pediatric patients after TOF repair [13]. The size of each branch pulmonary artery did not explain the difference in flow reversal. Harris and colleagues [14], confirming our findings of a greater RF in the LPA, found evidence that higher pulmonary vascular resistance (PVR) in the left lung may be responsible for the augmented diastolic flow reversal. However, the support for this mechanism was indirect and the etiology of a unilaterally elevated left-sided PVR remains unclear.

Based on these previous reports and our clinical observations, we hypothesized that compression of the left lung, as a result of cardiomegaly and rotation of the heart into the left chest, leads to greater LPA flow reversal. The objective of this study was to assess the relationship between heart size, mediastinal geometry and differential pulmonary regurgitation.

## Methods

We retrospectively analyzed the CMR studies performed in patients after TOF repair at our institution between June 2007 and November 2009. Patients with a RV to pulmonary artery conduit, status post stent implantation in the main pulmonary artery (MPA) or branch pulmonary arteries, more than mild discrete branch pulmonary artery narrowing defined by gradient >20 mmHg estimated via Doppler echocardiography, less than mild MPA regurgitation, defined as a regurgitant fraction (RF) <10 %, were excluded. Likewise, those with dextrocardia, congenital absence of the pulmonary valve, or a previous pulmonary valve replacement were excluded. The first CMR during the study period was included. For those patients who had undergone more than one CMR up until March 2016, the latest CMR study before pulmonary valve replacement was included as follow-up to delineate the change in geometric, volumetric, and flow parameters relative to the others. Clinical data including age at CMR, age at surgery, surgical procedure, were retrospectively collected from the patients’ medical records. This study was approved by the institutional research ethics board, and consent was waived.

### CMR

All patients underwent clinical CMR examinations at 1.5 T ('Avanto', Siemens Healthcare, Erlangen, Germany). The protocol consisted of scout imaging of the entire thorax in the axial plane using standard ECG gated steady state free precession (SSFP) imaging. A stack of SSFP short axis cine loops for ventricular volumetry and phase-contrast flow velocity mapping in the proximal RPA and LPA were obtained. Parameters of SSFP imaging were as follows: Flip angle 70°, parallel imaging with an acceleration factor of 2. Temporal resolution was adjusted to allow for 20 true reconstructed phases per cardiac cycle. For both SSFP and phase contrast imaging the in-plane spatial resolution was 1.5 mm × 1.5 mm and slice thickness 5 mm, minimal repetition and echo times. Other parameters of phase-contrast imaging were as follows: Flip angle 30°, 25 true phases per cardiac cycle.

Ventricular volumetry and phase contrast flow analysis were performed in the routine clinical fashion, using commercially available software (‘QMass Version 7.1’ and ‘QFlow Version 5.1’, Medis Medical Imaging Systems, Leiden, The Netherlands). Flow quantifications included forward flow volume; regurgitant flow volume; net flow volume (forward flow volume – regurgitant flow volume) and RF (forward flow volume divided by regurgitant flow volume, %).

### Geometric measurements

**Fig. 1:**
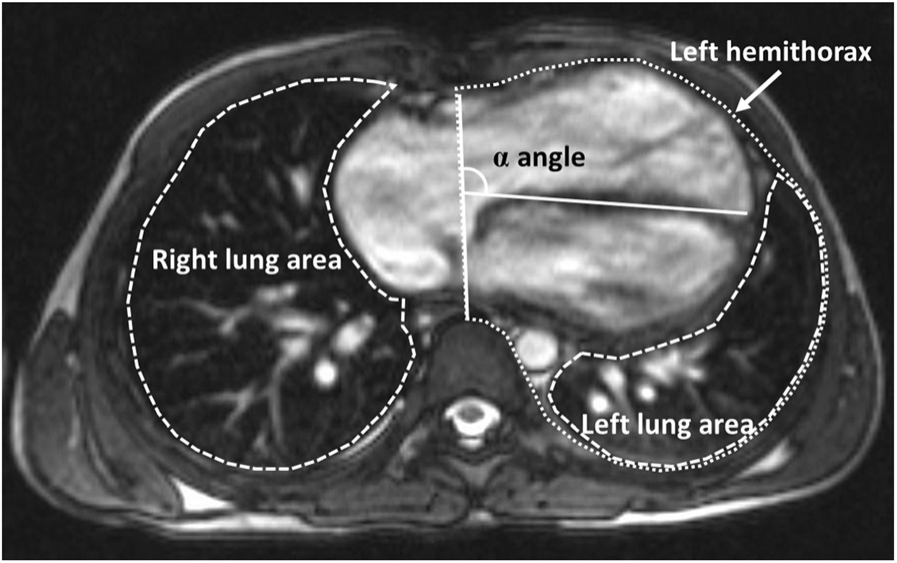
Axial plane scout image for cardiac and lung area measurements

All geometric measurements were made by a single reader (CD) on a ‘Centricity Enterprise’ (GE Healthcare, Piscataway, NJ) picture archiving and communication system (PACS) viewing station using the preset window settings. On the axial plane scout image (Fig. 1) with the largest cardiac surface area, the cardiac surface areas to the left and right of the midline as well as right and left lung surface areas were measured. ‘Lung area ratio’ was calculated as the quotient of lung area to the ipsilateral hemithorax area ratio. The cardiac angle (α angle) was measured between the thoracic anterior-posterior midline and the interventricular septum. In addition, the cross-sectional area (CSA) of the branch pulmonary arteries at peak systole (i.e. the largest area in the cardiac cycle) was measured on the magnitude images of the phase contrast acquisitions. All measurements other than ratios and angles were indexed to BSA (m^2^).

### Statistical analysis

Continuous variables are expressed as mean ± SD if normally distributed and otherwise as median and range. Categorical variables are given as counts and percentages of total. CMR parameters between left and right were compared using the Student’s *T*-test. Correlations were assessed using Pearson’s correlation analysis. In order to evaluate the association of a change in one parameter with the change in another parameter over time, the rate of change rate (Δ) was calculated as follows: Δ = (data at follow up – data at baseline) ÷ data at Baseline ÷ (interval between baseline and follow up). The method described by Bland and Altman was used to assess interobserver variability [15]. *P*-values of less than 0.05 were considered significant. Statistical analysis was performed using R software, version 3.2.2.

## Results

One hundred seventy eight CMR studies were performed in 141 patients during a 29-month period. The first diagnostic study per patient was included. Patients were excluded because of RV-to-pulmonary artery conduit (*n* = 23), insufficient data (*n* = 22), intravascular stents (*n* = 22), less than mild PR (*n* = 10), absent pulmonary valve syndrome (*n* = 8), status post pulmonary valve replacement (*n* = 3), significant pulmonary artery stenosis (*n* = 4), and dextrocardia (*n* = 1). Forty-eight studies were included in the analysis. Patient characteristics and the CMR results are summarized in Table 1. The median age at complete repair was 11.5 months (5 days – 5.6 years). Magnetic resonance imaging was undertaken at a mean age of 13.5 ± 2.9 years. Twenty-six patients (54 %) showed more than mild RV dilation (indexed right ventricular volume, or RVEDVi > 150 mL/m^2^). Mean RV ejection function was 48 ± 10 %. There were no patients with accidental significant pulmonary parenchymal issues, including lung hypoplasia, atelectasis, emphysema, or pleural effusion.

**Table 1:** Patient demographics (*n* = 48)

Characteristics	
Male, n (%)	22 (46 %)
Right aortic arch, n (%)	13 (27 %)
History of BT shunt, n (%)	10 (21 %)
Age at complete repair, median years (range)	0.95 (0.0–5.6)
Transannular patch repair, n (%)	35 (73 %)
CMR	
Age at CMR, years	13.5 ± 2.9
Body surface area, m^2^	1.5 ± 0.3
RVEDVi, mL/m^2^	170 ± 52
RVESVi, mL/m^2^	92 ± 41
RVEF, %	48 ± 10
LVEDVi, mL/m^2^	84 ± 15
LVESVi, mL/m^2^	36 ± 9
LVEF, %	58 ± 9
Heart rate at CMR, bpm	77 ± 15
QRS duration in ECG, millisecond	137 ± 24

*BT shunt* Blalock-Taussig shunt, *CMR* cardiac magnetic resonance, *ECG* electrocardiogram, *LVEDVi* BSA indexed left ventricular end-diastolic volume, *LVEF* left ventricular ejection fraction, *LVESVi* BSA indexed left ventricular end-systolic volume, *RVEDVi* BSA indexed right ventricular end-diastolic volume, *RVEF* right ventricular ejection fraction, *RVESVi* BSA indexed right ventricular end-systolic volume

### Right and left pulmonary artery flows

The flow data in the branch pulmonary arteries are summarized in Table 2. Total forward flow volume and net forward flow volume were smaller in the LPA than the RPA (2.49 ± 0.87 L/min/m^2^ vs 2.86 ± 0.89 L/min/m^2^; *p* = 0.02, 1.40 ± 0.51 vs 1.89 ± 0.60 mL/min/m^2^; *p* < 0.001, respectively). Regurgigtant fraction was greater in the LPA than the RPA (34 ± 10 % vs 43 ± 12 %; *p* < 0.001). There was no difference in regurgitant flow volume between the RPA and the LPA (*p* = 0.29). Right pulmonary artery CSA correlated with forward flow volume (*R* = 0.63, *p* < 0.001) and with regurgitant flow volume (*R* = 0.68, *p* < 0.001). Left pulmonary artery CSA correlated with regurgitant volume (*R* = 0.30, *p* = 0.04), but not with forward flow (*p* = 0.45).

**Table 2:** Student’s *t*-test comparison between the right and left parameters of cardiac magnetic resonance

	Right	Left	*p*-value
Proximal PA CSA, mm^2^/m^2^	225 ± 101	215 ± 71	0.87
Forward flow, L/min/m^2^	2.86 ± 0.89	2.49 ± 0.87	0.04
Reverse flow, L/min/m^2^	0.97 ± 0.43	1.08 ± 0.50	0.25
Net forward flow, L/min/m^2^	1.89 ± 0.60	1.40 ± 0.51	<0.001
Regurgitant fraction, %	34 ± 10	43 ± 12	<0.001
Lung area at cardiac level, cm^2^/m^2^	58.7 ± 7.9	32.0 ± 6.6	<0.001

*CSA* cross sectional area, *PA* pulmonary artery

### Differential pulmonary regurgitation and RV volume

**Fig. 2:**
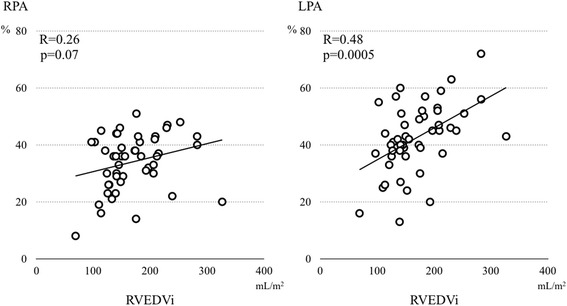
Correlation between regurgitant fraction and right ventricular end-diastolic volume. LPA, left pulmonary artery; RPA, right pulmonary artery; RVEDVi, indexed right ventricular end-diastolic volume

RVEDVi correlated with LPA RF (*R* = 0.48, *p* < 0.001), and a trend towards a correlation with RPA RF was present (*p* = 0.09; Fig. 2). RVEDVi correlated weakly with RPA regurgitant volume (*R* = 0.33, *p* = 0.02) and there was a trend towards a correlation with LPA regurgitant volume (*R* = 0.27, *p* = 0.06).

### Heart size and mediastinal geometry

**Fig. 3:**
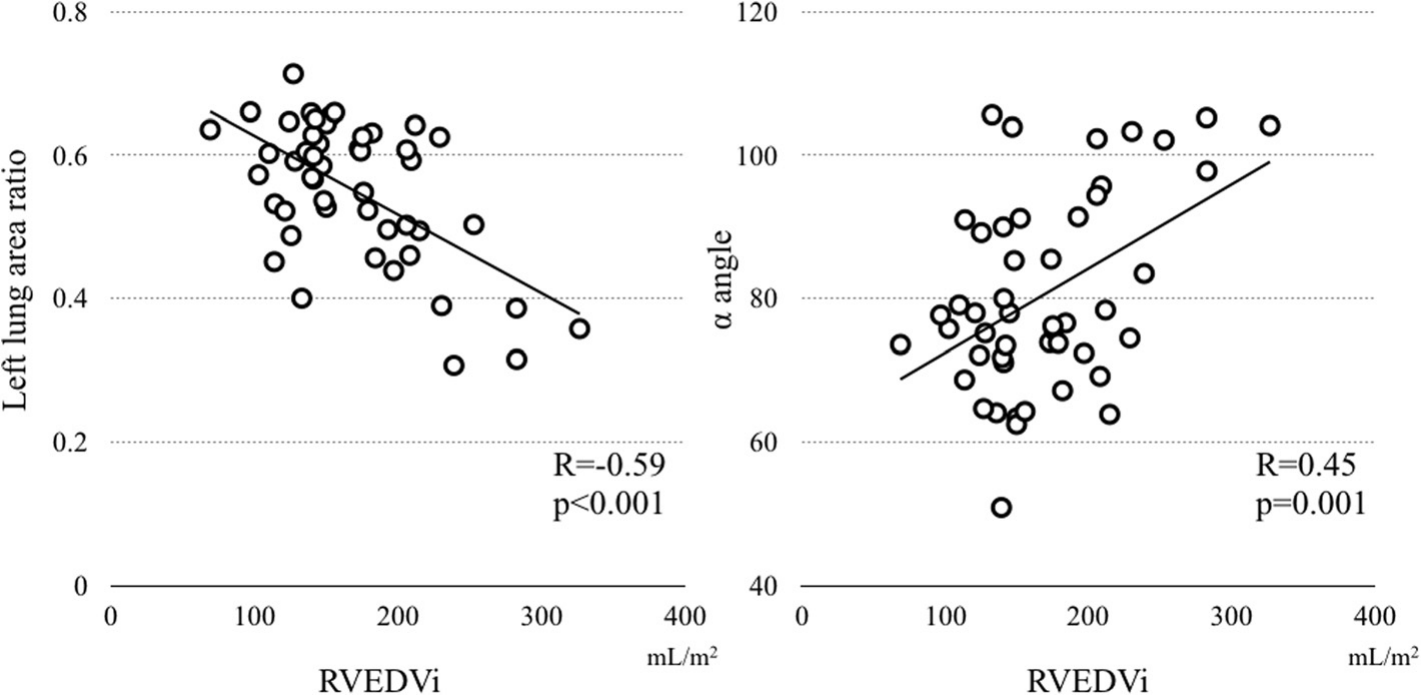
Correlation between right ventricular end-diastolic volume and left lung area ratio and cardiac axis. RVEDVi, indexed right ventricular end-diastolic volume

RVEDVi correlated with α angle (*R* = 0.45, *p* = 0.001), i.e. a larger RV was associated with an increased rotation of the heart to the left, and inversely with left lung area ratio (*R* = −0.59, *p* < 0.001; Fig. 3). These correlations were stronger in the subpopulation with an RVEDVi of more than 150 mL/m^2^ (α angle: *R* = 0.66, *p* < 0.001; left lung area ratio: *R* = −0.71, *p* < 0.001). RVEDVi correlated with indexed left ventricular end-diastolic volume (*R* = 0.66, *p* < 0.001).

### Differential pulmonary artery flows and lung size

**Fig. 4:**
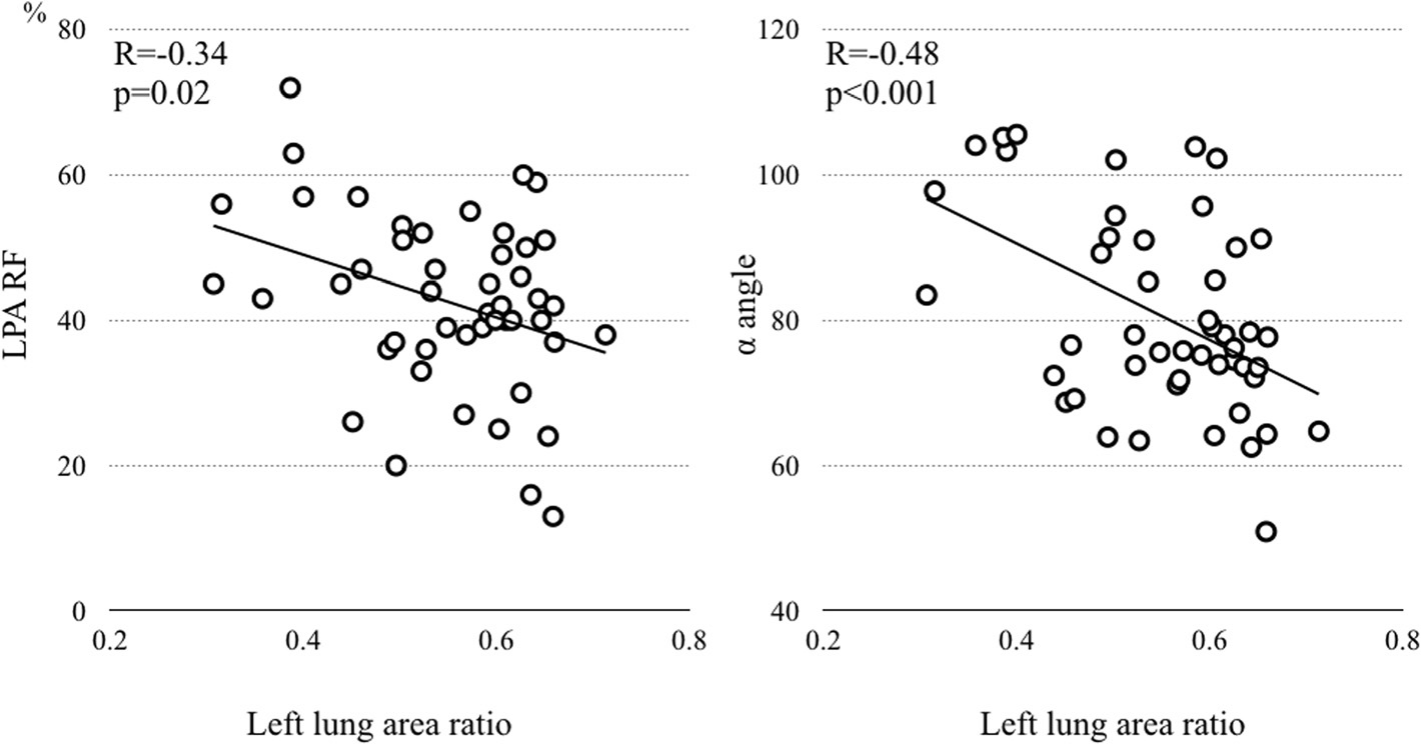
Correlation between left lung area ratio and left pulmonary artery regurgitant fraction and cardiac axis. LPA RF, regurgitant fraction in the left pulmonary artery

Left lung ratio correlated inversely with LPA RF (*R* = −0.34, *p* = 0.02) and α angle (*R* = −0.46, *p* < 0.001) (Fig. 4). Left lung ratio correlated with LPA net flow volume (*R* = 0.38, *p* = 0.006) There was no correlation between left lung ratio and LPA forward flow (*p* = 0.09) or LPA regurgitant volume (*p* = 0.9). RPA flow parameters were independent of left lung ratio.

### Follow-up CMR

Out of 48 patients with a baseline CMR, 20 patients (40 %) had follow up CMRs at a mean interval of 4.4 ± 2.1 years without interim pulmonary valve or branch pulmonary artery interventions. There was no significant difference in RVEDVi, LPA RF, RPA RF, α angle and left lung ratio between baseline and follow-up. The change (Δ) of α angle correlated with Δ RVEDVi (*R* = 0.51, *p* = 0.019) and Δ left lung ratio (*R* = −0.80, *p* < 0.001). Δ RVEDVi correlated inversely with Δ left lung ratio (*R* = −0.55, *p* = 0.012). Δ LPA RF correlated with Δ RVEDVi (*R* = 0.50, *p* = 0.03), whereas it did not correlate with Δ left lung ratio nor Δ α angle. Δ RVEDVi did not correlate with Δ RPA RF.

### Interobserver variability

**Fig. 5:**
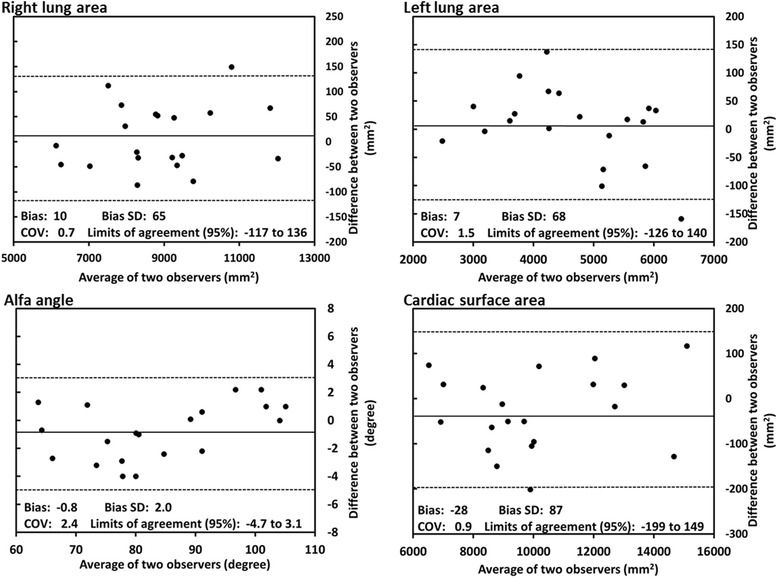
Bland-Altman plots for interobserver agreement of right lung area, left lung area, α angle and cardiac surface area measurements. COV, coefficient of variation

The interobserver agreements for area measurement of the lung and the heart as well as α angle were very good with COV ranging between 0.7 and 1.5 % and no significant bias (Fig. 5).

## Discussion

Pulmonary regurgitation is one of the most important hemodynamic long-term consequences after repair of TOF, causing RV dilation and dysfunction, which can result in exercise intolerance and fatal arrhythmia. The factors that drive PR, other than an incompetent pulmonary valve, are incompletely understood. Phase-contrast flow velocity mapping opened the door to the investigation of details of the volume and distribution of blood flow in the pulmonary arteries [16]. By using this technique, our previous study [13] provided clues to the pathophysiology of PR in patients after TOF repair, by showing that the relative amount of blood that streams back towards the heart is derived to a greater part from the LPA. Building on hypotheses generated on the basis of previous reports [14, 17–19], the key findings of this current study for better understanding of the mechanisms of PR after TOF repair are:

1. a smaller left lung size is associated with increased diastolic flow reversal in the LPA

and

2) right ventricular enlargement and rotation of the heart lead to compression of the left lung

In the present study, a smaller left lung area was associated with a greater RF in the LPA. Like any flow, pulmonary regurgitation is determined by a blood pressure gradient, in this case between the pulmonary artery and the RV during diastole. The majority of young patients after successful TOF repair can be assumed to have low RV diastolic pressures. Therefore, in the absence of pulmonary stenosis (which was an exclusion criterion in the present study), the primary determinant of diastolic flow reversal is the peripheral pressure in the lungs or, in the absence of shunts, the PVR [5]. Indeed, combining invasive manometry during cardiac catheterization and flows from CMR, Harris et al. [17] found an association between the pulmonary vascular resistance and flow reversal in the ipsilateral pulmonary artery. They concluded that the difference in pulmonary blood flow between the right and the left lung is a function of the differential PVR. In our cohort LPA size (in the absence of discrete obstruction) did not have a measurable impact on forward or reverse flow, supporting the concept that PVR and lung size, rather than central pulmonary artery size, are determinants of LPA blood flow. As the transpulmonary pressure gradient in these patients is usually small (<10 mmHg), relatively minor changes in PVR can, in principle, produce significant differences in PR flow [20, 21].

If PVR is indeed elevated in patients after TOF repair, why should the left lung be more affected than the right lung? Vosar et al. [19], who also documented a higher RF in the LPA as compared to the RPA after TOF repair, found a lower distensibility in the LPA as compared to the RPA. Lower distensibility of the central pulmonary arteries has been proposed as a marker of pulmonary arterial hypertension [22]. Other than the inherent characteristics of the LPA, there are no convincing risk factors for the development of pulmonary vascular disease in this cohort. Patients with unrepaired TOF patients typically have decreased pulmonary blood flow, hence, less risk to develop pulmonary vascular disease than congenital heart disease types with a left-to-right shunt. The arborization abnormalities in those with antegrade flow across their right ventricular outflow tract are typically mild. A histological study comparing the right and left lungs of patients with recently repaired TOF found similar sized arteries in both lungs [23]. Likewise, following ‘complete’ repair there are few factors that would explain intrinsic pulmonary vascular changes affecting more left than right. Overall, extrinsic compression is the most likely candidate mechanism for elevated left-sided PVR after in survivors after TOF repair.

Progressive enlargement of the right ventricle is typical in patients after TOF repair as a result of PR, tricuspid regurgitation, and RV dysfunction [5, 7, 8, 24]. In the current study we observed a correlation of RVEDVi with LPA RF and a trend towards an association with LPA regurgitant volume. LPA forward flow, as expected, was not associated with RVEDVi, confirming that volume loading through regurgitation is the principal driver of RV dilatation. As the central anteroposterior mediastinal space is limited between the sternum and the spine, the enlarging heart ‘bulges’ into the left chest, occupying an increasing portion of the left hemithorax, as evidenced by our results that RV enlargement is associated with a smaller left lung size. By the same mechanism, progressive right-sided cardiomegaly leads to a more leftward rotation of the heart, further compromising space for the left lung. These associations were more pronounced in patients with severely enlarged RVs (RVEDVi > 150 mL/m^2^), implying that there might be a threshold beyond which RV enlargement more significantly and directly impacts left lung volume. The mechanism of lung compression from enlarged hearts is well recognized in heart failure patients, in whom restrictive pulmonary physiology and reduced alveolar volume have been demonstrated [25, 26]. The results on follow-up support the existence of this connection between geometry, lung size and RV volume, by demonstrating an association between the rate of change in RV size with the change in cardiac levorotation and the progressive compression of the left lung.

### Limitations

Several limitations of this study warrant mention: Firstly, a larger cohort size than the present one may have unveiled additional associations. Secondly, cross-sectional lung area in a single-plane axial image was used as a surrogate for lung volume because lung segmentation was not feasible from the available images. Thirdly, pulmonary vascular resistance was not calculated, as invasive pulmonary artery pressure measurements were not widely available for this cohort. However, in the absence of branch pulmonary artery obstruction, LPA and RPA pressures are (nearly) identical and differences in PVR between the right and left lung will be solely the result of differences in blood flow which we captured. Therefore, no additional insight would have been gained from PVR measurements in this setting.

## Conclusions

### A positive feedback loop between progressive right ventricular dilatation and left pulmonary regurgitation?

**Fig. 6:**
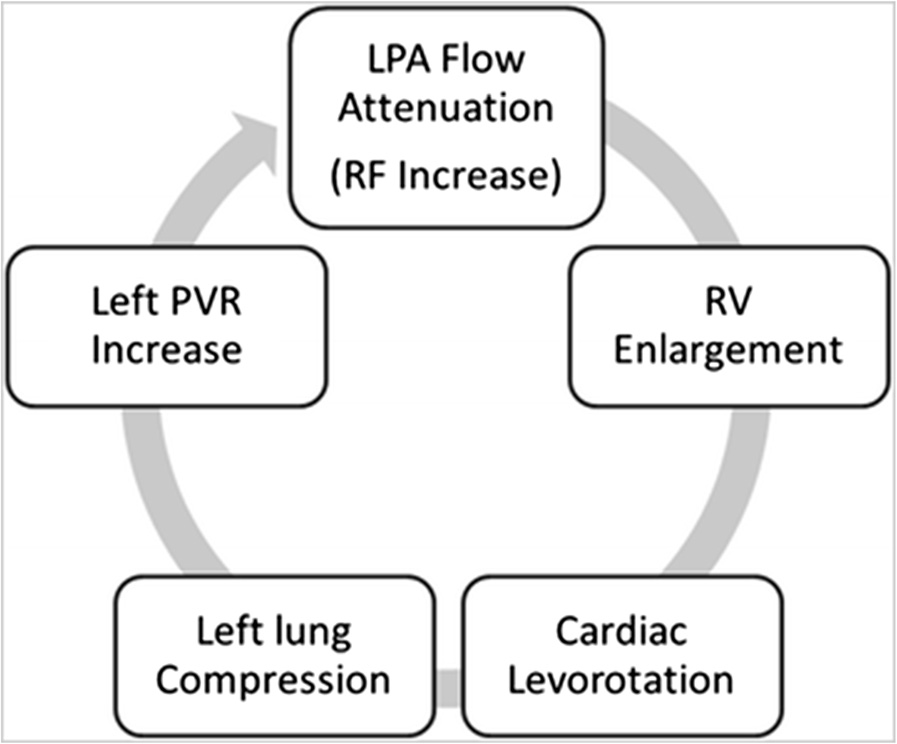
Hypothetical vicious circle involving mediastinal geometry, heart and lung sizes and pulmonary artery flow. LPA, left pulmonary artery; LV, left ventricle; PVR, pulmonary vascular resistance; RV, right ventricle

Based on the findings from the current and other studies the following scenario is plausible (Fig. 6): Volume loading from PR causes the RV to enlarge. Together with the LV that also dilates over time this results in cardiomegaly and rotation of the heart into the left hemithorax. Competing for the same finite space in the left chest, the heart compresses the left lung. Compression of the left lung elevates ipsilateral PVR. Elevated PVR attenuates the flow in the LPA, which in turn, raises the volume/pressure load on the RV, causing it to dilate further.

## Abbreviations

CMR, cardiovascular magnetic resonance; LPA, left pulmonary artery; MPA, main pulmonary artery; PR, pulmonary regurgitation; PVR, pulmonary vascular resistance; RF, regurgitant fraction; RPA, right pulmonary artery; RV, right ventricle; RVEDVi, indexed right ventricular end-diastolic volume; RVOT, right ventricular outflow tract; SSFP, steady state free precession; TOF, Tetralogy of Fallot.

## References

[1] Murphy JG, Gersh BJ, Mair DD, Fuster V, McGoon MD, Ilstrup DM, McGoon DC, Kirklin JW, Danielson GK (1993). Long-term outcome in patients undergoing surgical repair of tetralogy of Fallot. N Engl J Med.

[2] Gatzoulis MA, Balaji S, Webber SA, Siu SC, Hokanson JS, Poile C, Rosenthal M, Nakazawa M, Moller JH, Gillette PC and others. Risk factors for arrhythmia and sudden cardiac death late after repair of tetralogy of Fallot: a multicentre study. Lancet. 2000;356(9234):975–81. 10.1016/S0140-6736(00)02714-8 11041398

[3] Rowe SA, Zahka KG, Manolio TA, Horneffer PJ, Kidd L (1991). Lung function and pulmonary regurgitation limit exercise capacity in postoperative tetralogy of Fallot. J Am Coll Cardiol.

[4] Ghai A, Silversides C, Harris L, Webb GD, Siu SC, Therrien J (2002). Left ventricular dysfunction is a risk factor for sudden cardiac death in adults late after repair of tetralogy of Fallot. J Am Coll Cardiol.

[5] Redington AN (2006). Determinants and assessment of pulmonary regurgitation in tetralogy of Fallot: practice and pitfalls. Cardiol Clin.

[6] Wald RM, Redington AN, Pereira A, Provost YL, Paul NS, Oechslin EN, Silversides CK (2009). Refining the assessment of pulmonary regurgitation in adults after tetralogy of Fallot repair: should we be measuring regurgitant fraction or regurgitant volume?. Eur Heart J.

[7] d’Udekem Y, Ovaert C, Grandjean F, Gerin V, Cailteux M, Shango-Lody P, Vliers A, Sluysmans T, Robert A, Rubay J (2000). Tetralogy of Fallot: transannular and right ventricular patching equally affect late functional status. Circulation.

[8] Davlouros PA, Kilner PJ, Hornung TS, Li W, Francis JM, Moon JC, Smith GC, Tat T, Pennell DJ, Gatzoulis MA (2002). Right ventricular function in adults with repaired tetralogy of Fallot assessed with cardiovascular magnetic resonance imaging: detrimental role of right ventricular outflow aneurysms or akinesia and adverse right-to-left ventricular interaction. J Am Coll Cardiol.

[9] Niezen RA, Helbing WA, van der Wall EE, van der Geest RJ, Rebergen SA, de Roos A (1996). Biventricular systolic function and mass studied with MR imaging in children with pulmonary regurgitation after repair for tetralogy of Fallot. Radiology.

[10] Boechat MI, Ratib O, Williams PL, Gomes AS, Child JS, Allada V (2005). Cardiac MR imaging and MR angiography for assessment of complex tetralogy of Fallot and pulmonary atresia. Radiographics.

[11] Lewis MJ, O’Connor DS, Rozenshtien A, Ye S, Einstein AJ, Ginns JM, Rosenbaum MS (2014). Usefulness of magnetic resonance imaging to guide referral for pulmonary valve replacement in repaired tetralogy of Fallot. Am J Cardiol.

[12] Geva T (2011). Repaired tetralogy of Fallot: the roles of cardiovascular magnetic resonance in evaluating pathophysiology and for pulmonary valve replacement decision support. J Cardiovasc Magn Reson.

[13] Kang IS, Redington AN, Benson LN, Macgowan C, Valsangiacomo ER, Roman K, Kellenberger CJ, Yoo SJ (2003). Differential regurgitation in branch pulmonary arteries after repair of tetralogy of Fallot: a phase-contrast cine magnetic resonance study. Circulation.

[14] Harris MA, Weinberg PM, Whitehead KK, Fogel MA (2005). Usefulness of branch pulmonary artery regurgitant fraction to estimate the relative right and left pulmonary vascular resistances in congenital heart disease. Am J Cardiol.

[15] Bland JM, Altman DG (1986). Statistical methods for assessing agreement between two methods of clinical measurement. Lancet.

[16] Powell AJ, Maier SE, Chung T, Geva T (2000). Phase-velocity cine magnetic resonance imaging measurement of pulsatile blood flow in children and young adults: in vitro and in vivo validation. Pediatr Cardiol.

[17] Harris MA, Whitehead KK, Gillespie MJ, Liu TY, Cosulich MT, Shin DC, Goldmuntz E, Weinberg PM, Fogel MA (2011). Differential branch pulmonary artery regurgitant fraction is a function of differential pulmonary arterial anatomy and pulmonary vascular resistance. JACC Cardiovasc Imaging.

[18] Wu MT, Huang YL, Hsieh KS, Huang JT, Peng NJ, Pan JY, Huang JS, Yang TL (2007). Influence of pulmonary regurgitation inequality on differential perfusion of the lungs in tetralogy of Fallot after repair: a phase-contrast magnetic resonance imaging and perfusion scintigraphy study. J Am Coll Cardiol.

[19] Voser EM, Kellenberger CJ, Buechel ER (2013). Effects of pulmonary regurgitation on distensibility and flow of the branch pulmonary arteries in tetralogy of Fallot. Pediatr Cardiol.

[20] Ristow B, Ahmed S, Wang L, Liu H, Angeja BG, Whooley MA, Schiller NB (2005). Pulmonary regurgitation End-diastolic gradient is a Doppler marker of cardiac status: data from the heart and soul study. J Am Soc Echocardiogr.

[21] Hart S, Devendra G, Kim Y, Flamm S, Kalahasti V, Arruda J, Walker E, Boonyasirinant T, Bolen M, Setser R and others. PINOT NOIR: Pulmonic INsufficiency imprOvemenT with Nitric Oxide Inhalational Response. J Cardiovasc Magn Reson. 2013;15(1):75. 10.1186/1532-429X-15-75 PMC3844630 24006858

[22] Bogren HG, Klipstein RH, Mohiaddin RH, Firmin DN, Underwood SR, Rees RS, Longmore DB (1989). Pulmonary artery distensibility and blood flow patterns: a magnetic resonance study of normal subjects and of patients with pulmonary arterial hypertension. Am Heart J.

[23] Johnson RJ, Haworth SG (1982). Pulmonary vascular and alveolar development in tetralogy of Fallot: a recommendation for early correction. Thorax.

[24] Bokma JP, Winter MM, Oosterhof T, Vliegen HW, van Dijk AP, Hazekamp MG, Koolbergen DR, Groenink M, Mulder BJ, Bouma BJ (2015). Severe tricuspid regurgitation is predictive for adverse events in tetralogy of Fallot. Heart.

[25] Olson TP, Beck KC, Johnson BD (2007). Pulmonary function changes associated with cardiomegaly in chronic heart failure. J Card Fail.

[26] Agostoni P, Cattadori G, Guazzi M, Palermo P, Bussotti M, Marenzi G (2000). Cardiomegaly as a possible cause of lung dysfunction in patients with heart failure. Am Heart J.

